# Parenting style and young children's executive function mediate the relationship between parenting stress and parenting quality in two-child families

**DOI:** 10.1038/s41598-024-59225-x

**Published:** 2024-04-12

**Authors:** Guoying Qian, Bingbing Li, Lu Xu, Siqi Ai, Xin Li, Xueqing Lei, Gang Dou

**Affiliations:** 1 College of Preschool Education, Capital Normal University, Beijing, China; 2 School of Education, Hubei University of Arts and Science, 296 Longzhong Road, Xiangyang, Hubei China

**Keywords:** Parenting stress, Parenting quality, Parenting style, Executive function, Psychology, Psychology and behaviour, Quality of life

## Abstract

This study explored the relationship between parenting stress, parenting style, parenting quality, and young children's executive function. In total, 243 firstborns aged 2–9 years old (SD = 3.82) and their parents from two-child families in Beijing participated in the study, which used executive function tasks and parenting questionnaires. The results found that (1) parenting stress negatively predicted parenting quality; (2) parenting style partially mediated the relationship between parenting stress and parenting quality; (3) children's executive function partially mediated the relationship between parenting stress and parenting quality; and (4) the spoiled, democratic, permissive, and authoritarian parenting styles each play a chain mediating role with young children's executive function between parenting stress and parenting quality. Taken together, these findings provide implications for scientific parenting of children with different psychological characteristics (such as executive function) in multiple-child families under Parenting stress.

## Introduction

Parenting quality involves two important aspects: upbringing and education of children, including but not limited to looking after and feeding children, as well as cultivating their habits and promoting moral and emotional development, which all have significant impacts on their early development^1^. In recent years, the multiple adjustments of China's fertility policy have led to great changes in the country’s household population structure, allocation of urban and rural education resources, and people's lives^2^. Studies have shown that subsystems in the family microsystem (e.g., siblings, parent–child interactions, etc.) have experienced changes after the birth of second and third children, and these changes may have influence on the parenting quality and parent–child relationships in some families^3,4^. Previous studies have focused more on the impact of inter-parental relationships subsystems on sibling relationships subsystems^5^. According to family system theory multiple family subsystems interact with each other^6^, sibling subsystems and behaviors can also affect parental subsystems and behaviors. Therefore, this study systematically explores the four relationships among parenting stress, parenting style, executive function of young children and parenting quality in Chinese multi-child family.

Parenting stress refers to the pressure that parents experience while raising their children, which often leads to a sense of being unable to live a relaxed and enjoyable life^7^. For example, parents may feel inadequate in their parenting skills, doubt their ability to raise children, or experience excessive anxiety and fatigue during the process of raising their children. Parenting stress theory emphasizes that stressful events are common life events but may cause changes in the family social system^7^. From the perspective of this theory, the birth of second child is a stressful event for families. As the number of children grows, the economic and time costs of raising them will grow accordingly, thus increasing the burden on parents. However, considering the family microsystem^8^, although the birth of another child is a stressful event for families, as long as there is sufficient family support, psychological resources, or social support, and the parenting style is improved to cope with this stressful event, they can smoothly go through this special period.

Parenting stress is strongly related to parenting quality. Abidin^9^ suggested that parenting quality is associated with parenting stress and children's cognitive ability^10^. Knauer suggested that children's cognitive, social, and emotional development can be promoted in a warm, responsive, and encouraging parenting environment^11^. The parenting environment includes a relatively stable family natural environment and a dynamically developing social psychological environment. The former mainly refers to factors such as family geographical environment, daylighting, spatial layout, etc. The latter mainly refers to the education level, individual characteristics, parenting ideas and behaviors of family caregivers, and the relationships among family members. While the social psychological environment is considered a research priority of some critical value, certain psychological aspects like parenting stress have been neglected^12–14^. Therefore, we proposed the following:

### H1

Parenting stress has a negative predictive effect on parenting quality.

Parenting stress can not only directly affect parenting quality, but also indirectly affect parenting quality through mediating variables, such as parenting style and children's executive function^15–17^. Parenting style is a combination of daily parenting thoughts, behaviors, and emotional expressions in the process of child-rearing. Abidin proposed the parenting stress model, arguing that parenting stress affects parenting behavior^9^. Previous studies have identified parenting stress as an important factor influencing the parenting behavior of mothers with children aged 2 to 5 years. When mothers experience high levels of parenting stress, they tend to produce fewer positive parenting behaviors such as actively participating in their children's play and more negative parenting behaviors such as blaming their children^18^. Many families have become two-child families after the change in China’s fertility policy. This change has undoubtedly put pressure on parents in various ways, such as economical pressure and pressure to regulate sibling relationships, which may potentially compromise the parenting quality to a certain extent^19,20^.

In addition, Abidin^7^ pointed out that when parenting stress occurs, parents could exhibit inappropriate parenting styles^21^. He predicted that inappropriate parenting style would affect parenting quality. Moreover, he suggested that parenting style may mediate the relationship between parenting stress and parenting quality. Since parents are the primary caregivers in the parenting environment system, parenting quality is related to the parenting style, and parenting skills determine parenting quality to a certain extent^12^. After conducting a literature review, we proposed the following:

### H2

Parenting style mediates the relationship between parenting stress and parenting quality.

Executive function (EF) comprises higher cognitive functions, referring to a process in which integrated adaptations lead to achievement of predetermined goals in a flexible and appropriate manner^22^. Hughes^23^ found that EF can be divided into three sub-components: working memory, attentional flexibility, and inhibitory control. The EF of the firstborn child contributes positively to mental and physical health throughout the life cycle.

Previous studies have shown that fathers' parenting stress is often associated with various occupational stressors^24^. For the father, parenting stress can have a negative impact on several parenting dimensions. Increased stress predicts lower quality parent–child interactions^24^. Parenting stress also has a potential impact on child development, which includes effects on children's EF^22^. Previous studies have focused on the impact of parenting quality on children's EF^25,26^. However, parenting quality and children's EF influence each other; one study found that the development of EF in children aged of 36–60 months positively predicted parenting quality^27^. Therefore, we proposed the following:

### H3

Children’s EF partially mediates the relationship between parenting stress and parenting quality.

Consistent with systems theory, previous studies emphasized the transactional nature of parent–child behaviors across development^6,28,29^; parenting stress, parenting quality, and child development appear to mutually influence one another in families^30^. For example, mothers living with HIV face unique stressors impacting the interactions of parenting, parenting factors (e.g., communication skills) and child factors (e.g., emotion regulation)^31^. In a previous study, we examined the relationships between temperament, parenting style, and psychological adjustment among firstborns. The results showed that children’s temperament was significantly related to parenting style and psychological adaptation, and the parenting style was significantly related to psychological adaptation. The more a child's temperament leaned toward withdrawal, the more likely his parents would adopt an authoritarian parenting style, which might lead to the worse the child's psychological adaptation, and vice versa^32^.

Children's EF was found to be related to maternal parenting styles, with authoritative parenting positively predicting executive function problems and authoritarian parenting negatively predicting executive function problems. Parental discipline and control styles, responsiveness to the child, and support for children’s attempts at problem solving predict development of executive function^33^. Therefore, we proposed the following:

### H4

Parenting style and children's EF act as chain mediators between parenting stress and parenting quality.

## Method

### Participants

Purposive sampling of 243 firstborn children from two-child families in Beijing, China, was conducted, including 118 boys and 125 girls aged 2–9 years from kindergarten mostly. The mean age of firstborn children was 3.82 ± 1.14 years, and the age difference between the first- and second born children was 1–7 years. Parents’ age ranged from 26 to 56 years. The number of mothers and fathers with a college education was the largest, accounting for 75% and 75%; and family income with a total monthly income between 8000 and 12,000 yuan was the largest, accounting for 38.9%.

The study was reviewed and approved by the Research Ethics Committee of the first author's institution on 1st March, 2021(No. 2021003). Informed consent was obtained from all participating families in this study. All methods were performed in accordance with the relevant guidelines and regulations.

### Measures

#### Parenting stress index-short form (PSI-SF)

This study adopted the Parenting Stress Index-Short Form (PSI-SF) to understand parenting stress among parents of second children, and is the third version of Abidin’s^9^ parenting stress questionnaire. The form included 12 items (e.g., “After I have children, I can hardly do what I like”) completed by the parents of the child. Participants responded on a 5-point scale (1 = strongly disagree, 5 = strongly agree). Higher scores indicated that the parents had higher parenting stress. Cronbach’s α for PSI-SF was 0.812 in this study.

#### Parenting and family adjustment scale (PAFAS)

The Chinese version of the Parenting Behavior and Family Adjustment Scale^34^ was used to collect information on parenting quality in families with young children. The form included 26 items (e.g., “When a child behaves well, I will praise him or her.”) completed by the parents of the child. Items are rated on a 4-point scale (0 = *not at all consistent*, 3 = *fully consistent*), with higher scores indicating poorer parenting skills. Cronbach's α for the PAFAS was 0.904, and Cronbach's α for each latent variable met the basic criterion of being greater than 0.7.

#### Parenting style questionnaire

This study used the Parenting Style Questionnaire developed by Yang Lizhu^35^. The questionnaire included 40 items and classified parenting styles into five types: spoiled parenting (e.g., “Buy what the first-born children want”; Cronbach’s α = 0.825); democratic parenting (e.g., “Encourage the first-born children to do what they want to do”; Cronbach’s α = 0.833), permissive parenting (e.g., “Not caring about first-born children's wants and desires”; Cronbach’s α = 0.841), authoritarian parenting (e.g., “Beat or scold the first-born children when they disobey their parents”; Cronbach’s α = 0.877); inconsistent parenting (e.g., “Sometimes meet the first-born children’s unreasonable demands, and sometimes reject them”; Cronbach’s α = 0.867). Parents rated items on a 5-point scale (1 = never, 5 = always); the higher the score, the more prominent the type of parenting style.

#### Executive function measurement tools

Cronbach's α for executive function in young children was 0.889. The three experiments were conducted sequentially, and the formal testing time was controlled to be 30 min or less. The children were tested individually in the meeting rooms of their kindergartens or elementary schools.

##### Day-night stroop

Day-Night Stroop was developed by Gerstadt et al.^36^. The testing process was divided into two parts. The first part was a simple language naming task, which required young children to say "day" when they saw the "sun" image and "night" when they saw the "moon" image; The second part was the classic day and night task test, which was a day and night task test without nested rules. Children were required to say "night" when they saw the "sun" image and "day" when they saw the "moon" image. First, to ensure complete understanding before the formal test, the children were asked to practice three times, and the tester provided feedback; a child had to correctly complete a "day" and "night" test before the formal test could be administered; no feedback was provided in the formal test. The formal test comprised 16 trials (8 trials each for "day" and "night) in a randomized order. A score of 1 was given for correct responses and 0 for incorrect responses, with scores ranging from 0 to 16^36^.

##### Backward digit span

Backward digit span was developed by Carlson et al.^37^. The quiz materials used groups of numbers between 0 and 9. Before the test, a picture with numbers from 0 to 9 was presented to the children to ensure that they recognized the 10 numbers on the picture, and then three familiarization exercises were conducted. During the test, the number of digits in the group presented on the picture was incremented from 1 to 8, with the digits in each group being randomized. The tester first presented the child with a picture of numbers facing upwards and asked the subject to memorize as many number groups as possible, then the tester flipped the picture backward and asked the subject to report the number groups they had just memorized in the order of "back to front." The number of digits contained in the number group is used as the score for the backward number task (the difficulty of the number task increases sequentially, and no points are awarded for reporting errors in order or digits; the score is not cumulative). Each number group could be shown to the participant three times, and points were awarded for any one correct report of the number group^37^.

##### Dimensional change card sorting (DCCS)

DCCS was developed by Rao et al.^38^. The experimental material comprised 24 cards of red, yellow, and green triangles, circles and squares. There were 8 cards of each color, and the same number of each shape. Before beginning, the children were allowed to familiarize themselves with and identify the colors and shapes of the cards, and then three practice sessions were conducted. In the exercise, the tester first took out one card for each of the three shapes or colors and asked the children to find all the other cards of the same shape or color for classification. The children passed all three exercises before conducting the formal experiment. In the formal experiment, eight color and shape classifications were required in each of the ABBA balance orders. For each dimensional classification, children scored 1 point for all correct responses and 0 points for incorrect ones^39^.

After all EF tasks were completed and scores were recorded, the scores were averaged and total EF scores were calculated to represent each child’s EF level.

### Procedure

Mothers of two-child families were first purposefully selected and given questionnaires. This allowed the researcher to understand their basic situation as well as to make a comprehensive assessment of the data. Next, the firstborn children took the EF test. Before conducting the EF test, the mothers were interviewed to determine that the toddlers had no developmental or language disorders, intellectual disability, color blindness, or color deficiency problems. The EF test consisted of three experiments, which were conducted sequentially. The formal testing time was controlled to 30 min or less. The children were tested one at a time to ensure that they successfully completed the EF tasks.

### Data analysis

Before the formal analysis of the data, a common method bias test was conducted using the Harman one-way test. Then two-by-two relationship between the four variables was analyzed using Pearson's product-difference correlation with IBM SPSS Statistics for Windows, version 22.0 (https://www.ibm.com/cn-zh/spss); the PROCESS plug-in (Model 4) in the same software and selected the bootstrap method to analyze the mediation effect of parenting style between parenting stress and parenting quality and the mediation effect of firstborn toddler executive function between parenting stress and parenting quality. Finally, the PROCESS plug-in (Model 6) was used to analyze the chain mediating role of parenting style and firstborn executive function in the relationship between parenting stress and parenting quality.

### Ethics declaration

The studies involving human participants were reviewed and approved by the Scientific Research Ethics Committee of College of Preschool Education, Capital Normal University. All procedures performed in studies involving human participants were in accordance with the ethical standards of the institutional research committee and with the 1964 Helsinki Declaration and its later amendments. Informed consent was obtained from parents of children.

## Results

### Analysis of common methodological bias

Data for the study were obtained from self-reports, which can lead to common method bias. Therefore, a common method bias test was conducted using the Harman one-way test. All topics of the four variables, including parenting stress, parenting style EF and parenting quality, were analyzed; factor analysis showed that the variance explained by the first common factor was 22.73%, which is much less than the critical value of 40%. In addition, there were 11 factors with a larger feature weight than 1, indicating that there was no significant bias in the common method in this study.

### Correlation analysis

Pearson's correlation analysis was used to analyze the correlations of four variables: parenting stress with the second child, the five types of parenting style (spoiled, democratic, permissive, authoritarian, and inconsistent), and EF and parenting quality in the first child.

Table 1 showed that parenting stress was positively associated with the spoiled, permissive, authoritarian, and inconsistent parenting styles. Parenting stress was negatively correlated with democratic parenting style, firstborn’s EF, and parenting quality.

**Table 1:** Results of correlation analysis of each variable.

	*M*	*SD*	1	2	3	4	5	6	7	8
1 Parenting Stress	2.585	0.279	1							
2 Spoiled	1.614	0.430	0.352**	1						
3 Democratic	3.352	0.323	− 0.451**	− 0.524**	1					
4 Permissive	1.936	0.447	0.469**	0.601**	− 0.511**	1				
5 Autocratic	2.190	0.430	0.331**	0.388**	− 0.524**	0.611**	1			
6 Inconsistent	2.013	0.509	0.149**	0.431**	− 0.394**	0.371**	0.598**	1		
7 Firstborn executive function	28.020	4.901	− 0.315**	− 0.268**	0.369**	− 0.411**	− 0.293**	− 0.424**	1	
8 Parenting quality	2.299	0.168	− 0.417**	− 0.322**	0.218**	− 0.387**	− 0.329**	− 0.105*	0.382**	1

*p < 0.05, **p < 0.01.

Spoiled, permissive, authoritarian, and inconsistent parenting styles were all negatively associated with EF and parenting quality of firstborns. However, democratic parenting style was positively associated with executive function and parenting quality of firstborns, and executive function and parenting quality of firstborns were positively associated.

### Mediation effect analysis

#### Mediation effect of parenting style between parenting stress and parenting quality

This study used the PROCESS plug-in (Model 4) in SPSS software and selected the bootstrap method for direct and mediated effects testing. The sample size was set to 5000 iterations, and the confidence interval was set to 95%. Table 2 illustrated the mediating effect of parenting style between parenting stress and parenting quality.

**Table 2:** Mediation effect analysis of spoiled, democratic, permissive, authoritarian, and inconsistent parenting styles in the relationship between parenting stress and parenting quality.

Predictive variables	Result variables	*R*^*2*^	*F*	*β*	*SE*	*t*
Parenting stress	Parenting quality	0.487	23.237	− 0.342	0.038	− 8.757***
Parenting stress	Spoiled	0.332	19.110	0.234	0.024	5.704***
Parenting stress	Parenting quality	0.535	35.286	− 0.216	0.031	− 3.906***
Spoiled				− 0.287	0.022	− 5.364***
Parenting stress	Democratic	0.375	22.147	− 0.318	0.038	− 5.574***
Parenting stress	Parenting quality	0.296	18.354	− 0.267	0.049	− 4.752***
Democratic				0.225	0.029	4.321***
Parenting stress	Permissive	0.299	17.642	0.242	0.065	5.327***
Parenting stress	Parenting quality	0.332	21.586	− 0.301	0.034	− 6.292***
Permissive				− 0.189	0.072	− 3.064**
Parenting stress	Authoritarian	0.455	20.557	0.319	0.055	7.364***
Parenting stress	Parenting quality	0.281	11.239	− 0.288	0.037	− 5.444***
Authoritarian				− 0.352	0.082	− 9.085**
Parenting stress	Inconsistent	0.221	5.764	0.115	0.046	2.117*
Parenting stress	Parenting quality	0.308	7.692	− 0.337	0.085	− 7.589***
Inconsistent				− 0.063	0.074	− 1.195

*p < 0.05, **p < 0.01, ***p < 0.001.

Table 2 showed that parenting stress had a significant negative predictive effect on parenting quality (β = − 0.342, P < 0.001). It also had a significant positive predictive effect on the spoiled (β = 0.234, P < 0.001), permissive (β = 0.242, P < 0.001), authoritarian (β = 0.319, P < 0.001), and inconsistent parenting styles (β = 0.115, P < 0.05). However, parenting stress had a significant negative predictive effect on democratic parenting style (β = − 0.318, P < 0.001).

Spoiled (β = − 0.287, P < 0.001), permissive (β = − 0.189, P < 0.01), and authoritarian (β = − 0.352, P < 0.01) parenting styles all had a significant negative predictive effect on parenting quality. Democratic parenting style had a significant positive predictive effect on parenting quality (β = 0.225, P < 0.001), and inconsistent parenting did not have a significant predictive effect on parenting quality (β = − 0.063, P > 0.05).

When Parenting stress and spoiled, democratic, permissive, and authoritarian parenting styles acted simultaneously on parenting quality, the effect coefficient of the independent variable was significantly lower, indicating the existence of a mediating effect; the addition of the mediating variables spoiled, democratic, permissive, and authoritarian parenting styles still significantly affected parenting quality, indicating that these styles partially mediated the effect between Parenting stress and parenting quality. Since inconsistent parenting style did not meet the prerequisite conditions for the mediating effect test, indicating that it did not play a significant mediating role between parenting stress and parenting quality, its mediating effect will not be tested.

#### Mediation effect analysis of firstborn toddler executive function between parenting stress and parenting quality

This study used the PROCESS plug-in (Model 4) in SPSS and selected the bootstrap method for direct and mediated effects testing. The sample size was set to 5000 iterations, and the confidence interval was set to 95%. Table 3 showed the mediating effect of firstborn EF between parenting stress and parenting quality.

**Table 3:** Mediation Effect Analysis of firstborn children's executive function between parenting stress and parenting quality.

Predictive variables	Result variables	*R*^*2*^	*F*	*β*	*SE*	*t*
Parenting stress	Parenting quality	0.487	23.237	− 0.342	0.038	− 8.757***
Parenting stress	Firstborn executive function	0.341	19.411	− 0.402	0.037	− 9.009***
Parenting stress	Parenting quality	0.258	15.276	− 0.271	0.025	− 6.322***
Firstborn executive function				0.319	0.076	7.851***

*p < 0.05, **p < 0.01, ***p < 0.001.

Parenting stress had a significant negative predictive effect on parenting quality (β = − 0.342, P < 0.001) and firstborn child EF (β = − 0.402, P < 0.001), and a significant positive predictive effect of firstborn child EF on parenting quality (β = 0.319, P < 0.001). When parenting stress and firstborn EF acted simultaneously on parenting quality, the effect coefficient of the independent variable was significantly lower, indicating a mediating effect; the addition of firstborn EF still significantly affected parenting quality, suggesting that firstborn EF partially mediated the effect between parenting stress and parenting quality.

#### The chain mediation effect of parenting style and firstborn executive function in the relationship between parenting stress and parenting quality

By testing the hypothesized relationships between Parenting stress, parenting style, firstborn EF, and parenting quality, the final results showed that there was no direct role of inconsistent parenting style and parenting quality, nor was there a mediating role between Parenting stress and parenting quality. In contrast, spoiled, democratic, permissive, and authoritarian parenting styles and firstborn EF played a significant mediating role between parenting stress and parenting quality. Therefore, a test was conducted to verify whether there was a chain mediation role of parenting style (spoiled, democratic, permissive, and authoritarian) and firstborn EF in the relationship between parenting stress and parenting quality. We used the PROCESS plug-in (Model 6) in SPSS software to test for chain mediation effects, using the bootstrap method with 5000 samples. The chain mediating effect of spoiled, democratic, permissive, and authoritarian parenting styles and firstborn EF between parenting stress and parenting quality was significant (Table 4).

**Table 4:** Analysis of the chain mediation effect of parenting style and firstborn toddler executive function in the relationship between parenting stress and parenting quality.

Predictive variables	Result variables	*R*^*2*^	*F*	*β*	*SE*	*t*
Parenting stress	Firstborn executive function	0.341	19.411	− 0.402	0.037	− 9.009***
Parenting stress	Spoiled	0.332	19.110	0.234	0.024	5.704***
Spoiled	Firstborn executive function	0.288	15.324	− 0.188	0.058	− 2.897**
Parenting stress	Parenting quality	0.395	21.249	− 0.118	0.032	− 2.145*
Spoiled				− 0.210	0.039	− 3.285**
Firstborn executive function				0.231	0.066	3.990***
Parenting stress	Democratic	0.268	9.858	− 0.221	0.028	− 4.203***
Democratic	Firstborn executive function	0.232	6.296	0.196	0.037	2.669**
Parenting stress	Parenting quality	0.285	11.337	− 0.134	0.063	− 2.145*
Democratic				0.229	0.089	4.285***
Firstborn executive function				0.208	0.067	2.990**
Parenting stress	Democratic	0.268	9.858	− 0.221	0.028	− 4.203***
Parenting stress	Permissive	0.258	12.856	0.211	0.045	3.842**
Permissive	Firstborn executive function	0.199	6.228	− 0.166	0.078	− 2.997**
Parenting stress	Parenting quality	0.276	15.285	− 0.152	0.044	− 3.145**
Permissive				− 0.244	0.059	− 4.184***
Firstborn executive function				0.216	0.061	3.998***
Parenting stress	Authoritarian	0.274	9.364	0.199	0.033	2.715**
Authoritarian	Firstborn executive function	0.185	4.191	− 0.167	0.076	− 2.997**
Parenting stress	Parenting quality	0.242	8.287	− 0.114	0.079	− 2.111*
Authoritarian				− 0.236	0.034	− 4.184***
Firstborn executive function				0.202	0.055	3.057**

*p < 0.05, **p < 0.01, ***p < 0.001.

## Discussion

In this study, we explored the relationship between parenting stress, parenting style, parenting quality, and young children's executive function. The results showed that spoiled, democratic, permissive, authoritarian parenting styles, and EF of young children had a chain mediation effect between parenting stress and parenting quality.

Parenting stress was found to have a negative predictive effect on parenting quality in this study. It has been noted that parenting stress in two-child families is significantly higher than that in one-child families^19,40^, especially for mothers, who face higher parenting stress in general; demographic variables such as birth order, gender combination, and age gap have significant effects on mothers' stress levels in two-child families. Our results are consistent with Abidin’s viewpoint suggested that parenting quality is associated with parenting stress^9^, which can be explained by parenting stress theory and family system theory. Parenting stress theory emphasizes that the birth of second child is a stressful life event for families. Raising a child requires a lot of scarce resources in the family, such as human resources, time and money. As the number of children increases, the economical and time cost of childrearing rises. With the total amount of family resources remaining basically unchanged, the first child will inevitably get less parental care than before, resulting in a lower parenting quality. Meanwhile, according to the spill-over effect of family system theory, parenting stress and possibly negative emotions in the parental subsystem will overflow into the parent–child subsystem, thereby affecting the quality of parenting.

From the present results, parenting stress can affect parenting quality both directly and indirectly through the effects of parenting style (spoiled, democratic, permissive, and authoritarian), which is consistent with previous research^41^. Studies have shown that parenting stress can lead parents to prefer negative parenting styles and undermine their ability to use positive parenting methods, thus affecting parenting quality. For example, parents with higher parenting stress are less likely to exhibit warm parenting behaviors in parent–child interactions^42^ and more likely to overreact or engage in harsh parenting practices^43^. Abidin's^9^ theoretical model also suggests that parenting stress leads parents to develop negative parenting styles. When parents experience such stress, they become irritable and can no longer deal calmly with their young children's behavioral problems or adopt appropriate parenting styles; they may even choose to overlook their children's problems and provide children with less attention in order to take a break and alleviate their own stress. This can eventually lead to poor parent–child relationships, poor parenting outcomes, and reduced parenting quality^44^.

The present results indicated that parenting stress affects parenting quality through the mediating role of their children’s EF. There is evidence that parenting stress is negatively associated with children's false beliefs and that parents with relatively low levels of stress may be able to engage in parent–child interactions that promote children's theory of mind, while parents with high levels of stress do the opposite^42^. Studies have found that if adults provide scaffolding for children, children can construct their own thinking, thus reaching a new level of developmental function. Mothers’ affectionate care, encouragement and support are all beneficial for the development of children's thinking^45^. When parents themselves are in a good state and do not feel Parenting stress, they are more likely to exhibit the aforementioned behaviors, thereby improving children's abilities and achieving good parenting quality. On the contrary, it may lead to low self-regulation ability of young children, which in turn affects the quality of parenting. Researchers have found that children with lower EF are more likely to show behavioral problems, such as anxiety withdrawal, anger aggression, and low social competence^46,47^. Moreover, longitudinal studies on parenting stress and internalizing problems in young children have shown that parenting stress is associated with children’s internalizing problems when they grow up, i.e., parenting stress may lead to psychological problems such as anxiety and depression in young children^48^.

Parenting stress also affects parenting quality through parenting style and then through young children’s EF. That is, parenting stress can affect parenting quality directly, and also indirectly through parenting styles and young children’s EF. This result is consistent with previous studies showing that stressed parents are more inclined to choose authoritarian parenting styles, leading to an increase in implicit and explicit behavioral disorders^49^. A study by Davis and Carter^50^ showed that mothers experience significantly more stress in parenting than fathers. Thus, maternal parenting stress negatively predicted maternal warmth behavior after one year and had an indirect effect on children's sensitive cooperation and anger aggression^20^. The reason might be that higher or lower parenting stress can lead to positive or negative parenting styles that affect children's social adjustment. On the one hand, positive parenting styles can promote children's language development, parent–child attachment, and parent–child interaction. On the other hand, children's good language ability, close parent–child attachment, and effective parent–child support strategies are all factors that promote children's executive function. Therefore, it is easy to see that when parenting stress is relatively low, parents are more inclined to adopt parenting styles that favor acceptance, support, encouragement, and sensitivity, which can positively promote all aspects of children's competence. Simultaneously, children are also more willing to accept and internalize the demands and expectations of parents who are sensitive, tolerant, and gentle toward them, positively affecting parenting quality. Conversely, when parenting stress is excessive, parents are more likely to adopt a parenting style that favors rejection, exclusion, and punishment, thereby inhibiting children’s healthy development. Children also tend to develop a sense of resistance and rebellion against authoritarian, arbitrary, and rejecting parental instructions and demands, thus negatively influencing parenting quality^51^.

Previous studies have focused more on the impact of parental factors on children's development, without paying attention to whether children's development can in turn affect parents' parenting quality. Our study not only found that parenting stress and parenting style affect children's executive function, but also further found that children's executive function can also affect parents' parenting quality, further supporting family system theory, which suggests multiple family subsystems interact with each other^6,28,29^, sibling subsystems and behaviors can also affect parental subsystems and behaviors.

The present study is limited in several ways that may provide directions for future research. First, the design of our study was cross-sectional, and only one measure each was administered to parents and children. Although our findings suggest that parenting stress is closely related to parenting quality, there is a lack of further exploration of the interaction mechanism involved, and the same problem exists in the mediating mechanisms of parenting style and children's EF between parenting stress and parenting quality. Future studies could further track the relationship between parenting styles and firstborn’s EF in terms of parenting stress and parenting quality. Second, parenting quality was examined from the parenting perspective, and was not assessed from the perspective of the children’s development, future research needs to use a more comprehensive approach to reflect parenting quality. Third, this study mainly investigated mothers of firstborn children, in the future, there is a need to investigate fathers and mothers separately and compare the differences. Finally, due to the fact that many first-borns' younger sister or brother are too young to successfully complete the relevant tasks, their executive functions have not been evaluated in our study, even without considering that other family factors may be more relevant or act as moderators (e.g., poverty, social support), which should be studied in the future.

## Conclusion

In summary, this study found that parenting stress had a negative predictive effect on parenting quality; democratic, spoiled, permissive, and authoritarian parenting styles partially mediated the relationship between parenting stress and parenting quality. Early childhood EF partially mediated the relationship between parenting stress and parenting quality. Finally, the spoiled, democratic, permissive, and authoritarian parenting styles and young children's EF play a chain mediating role between parenting stress and parenting quality (Supplementary information).

### Supplementary Information

Supplementary Information.

## Data Availability

The data that support the findings of this study are available from the corresponding author upon reasonable request.
