# Supplementary material for: Parenting style and young children's executive function mediate the relationship between parenting stress and parenting quality in two-child families
Source: Sci Rep. 2024 Apr 12;14:8503. doi: 10.1038/s41598-024-59225-x (PMC11009342; doi:10.1038/s41598-024-59225-x)
Supplement: Supplementary file 1 — Supplementary Information. [file 41598_2024_59225_MOESM1_ESM.docx]

Three questionnaires

| Parenting Stress Questionnaire | | | | | | |
| --- | --- | --- | --- | --- | --- | --- |
| serial number | title | strongly agree | agree | inconclusive | disagree | Strongly disagree |
| 1 | I often feel unable to get things right. |  |  |  |  |  |
| 2 | I have sacrificed much of my own life to meet the needs of this child which I did not expect. |  |  |  |  |  |
| 3 | Since having children, I've felt bound by the responsibilities of parenthood. |  |  |  |  |  |
| 4 | Since having kids, I can't try new and different things. |  |  |  |  |  |
| 5 | I can barely do the things I love since having this baby. |  |  |  |  |  |
| 6 | I feel regret or unhappiness after buying clothes for myself. |  |  |  |  |  |
| 7 | There are so many things in my life that bother me. |  |  |  |  |  |
| 8 | I had no idea that the birth of this child would cause so many problems between us as a couple. |  |  |  |  |  |
| 9 | I felt lonely and friendless. |  |  |  |  |  |
| 10 | When I see a party, I usually don't have a good time. |  |  |  |  |  |
| 11 | I don't enjoy socializing with people as much as I used to. |  |  |  |  |  |
| 12 | I don't appreciate things and enjoy life as much as I used to. |  |  |  |  |  |

| Parenting Style Questionnaire | | | | | |
| --- | --- | --- | --- | --- | --- |
| serial number | title | never | rarely | sometimes | always |
| 1 | Not criticizing your child for fear of being upset or making a mistake. |  |  |  |  |
| 2 | Unrealistic praise for children. |  |  |  |  |
| 3 | Kids can do whatever they want. |  |  |  |  |
| 4 | I'll do whatever it takes to make my kids happy. |  |  |  |  |
| 5 | No right and wrong with children. |  |  |  |  |
| 6 | Buy your kids whatever they want. |  |  |  |  |
| 7 | Oversensitive to the slightest abnormality in the child. |  |  |  |  |
| 8 | Encourage your child to do what he would do. |  |  |  |  |
| 9 | Help your child when he or she is having difficulty in learning or doing other things. |  |  |  |  |
| 10 | Cultivate your child's speciality according to his or her own interests. |  |  |  |  |
| 11 | Obedience through reasoning. |  |  |  |  |
| 12 | Allow your child to interrupt and ask questions when talking to them. |  |  |  |  |
| 13 | Amusement and games with children. |  |  |  |  |
| 14 | When a child does something wrong, ask why before criticizing. |  |  |  |  |
| 15 | Provide serious answers to the child's questions. |  |  |  |  |
| 16 | Praise or reward children in appropriate ways. |  |  |  |  |
| 17 | When instructing a child to do something, make sure the child understands why or how to do it. |  |  |  |  |
| 18 | Not caring about the little things in a child's life. |  |  |  |  |
| 19 | Not paying attention to what the child is doing or how they are doing it. |  |  |  |  |
| 20 | Ignoring your child's questions when you're busy yourself. |  |  |  |  |
| 21 | It doesn't matter if your child obeys you or not. |  |  |  |  |
| 22 | Doesn't care if your child makes a mistake. |  |  |  |  |
| 23 | Lack of knowledge of the exact amount of time the child is not with the parents. |  |  |  |  |
| 24 | Make no promises to your child. |  |  |  |  |
| 25 | Children just do their own thing at home without specific requests from parents. |  |  |  |  |
| 26 | No punishment or reward for children. |  |  |  |  |
| 27 | Do for your child what he can't do well. |  |  |  |  |
| 28 | Watching your child do things and always pointing. |  |  |  |  |
| 29 | Children are impatient with questions. |  |  |  |  |
| 30 | It's up to the parents to decide which area of speciality they want to develop in their children. |  |  |  |  |
| 31 | Scolding children when they disobey their parents. |  |  |  |  |
| 32 | Parental consent is required for who the child stays with. |  |  |  |  |
| 33 | Require children to report everything they do to their parents. |  |  |  |  |
| 34 | Asking a child to do something without giving reasons or how to do it. |  |  |  |  |
| 35 | Sometimes it's okay to criticize and sometimes it's okay for your child to do something wrong. |  |  |  |  |
| 36 | Sometimes caring and sometimes not caring about the child's learning and life. |  |  |  |  |
| 37 | Sometimes the child's crying is identified and sometimes it is forced. |  |  |  |  |
| 38 | Sometimes convincing children, sometimes forcing them. |  |  |  |  |
| 39 | Sometimes fulfilling and sometimes refusing the child's unreasonable demands. |  |  |  |  |
| 40 | The same thing, sometimes allowed, sometimes denied. |  |  |  |  |

| **Parenting and Family Adaptation Scale** | | | | | |
| --- | --- | --- | --- | --- | --- |
| serial number | title | not at all | a bit consistent | sometimes consistent | fully consistent |
| 1 | If a child doesn't do what I want, I will give up and make things right myself. |  |  |  |  |
| 2 | I will offer rewards or fun activities for good behavior. |  |  |  |  |
| 3 | When a child misbehaves, I apply some sort of threat (e.g. turning off the TV) but do not act on it. |  |  |  |  |
| 4 | I shout or get angry with my child when he/she misbehaves. |  |  |  |  |
| 5 | I praise my child for good behavior. |  |  |  |  |
| 6 | When misbehaving, I try to make the child feel bad (e.g., guilt, shame) so as to teach him/her a lesson. |  |  |  |  |
| 7 | I give attention such as hugs, winks, smiles and kisses when the child is well-behaved. |  |  |  |  |
| 8 | I will hit my child when he/she misbehaves (e.g. spanking). |  |  |  |  |
| 9 | I argue with my child about his/her behavior and attitude. |  |  |  |  |
| 10 | I give my child what he/she wants when he/she is angry or irritable. |  |  |  |  |
| 11 | I talk/talk to my child. |  |  |  |  |
| 12 | I like to give my children hugs, kisses and snuggles. |  |  |  |  |
| 13 | I'm proud of my kids. |  |  |  |  |
| 14 | I like spending time with my kids. |  |  |  |  |
| 15 | I have a good relationship with my children. |  |  |  |  |
| 16 | I feel happy. |  |  |  |  |
| 17 | I feel sad or depressed. |  |  |  |  |
| 18 | I'm happy with my life. |  |  |  |  |
| 19 | I can cope with the negative emotions that come with raising a child. |  |  |  |  |
| 20 | Our family members help and support each other. |  |  |  |  |
| 21 | Our family members get along well. |  |  |  |  |
| 22 | Our family members fight or argue. |  |  |  |  |
| 23 | Our family members blame or belittle each other. |  |  |  |  |
| 24 | My spouse and I work as a team when it comes to parenting. |  |  |  |  |
| 25 | I don't agree with my spouse when it comes to parenting. |  |  |  |  |
| 26 | I have a good relationship with my spouse. |  |  |  |  |
